# Genetic profile of a large Spanish cohort with hypercalcemia

**DOI:** 10.3389/fendo.2024.1297614

**Published:** 2024-03-22

**Authors:** Alejandro García-Castaño, Leire Madariaga, Sara Gómez-Conde, Pedro González, Gema Grau, Itxaso Rica, Gustavo Pérez de Nanclares, Ana Belén De la Hoz, Aníbal Aguayo, Rosa Martínez, Inés Urrutia, Sonia Gaztambide, Luis Castaño

**Affiliations:** ^1^Biobizkaia Health Research Institute, Hospital Universitario Cruces, CIBERDEM, CIBERER, EndoERN, Barakaldo, Bizkaia, Spain; ^2^Pediatric Nephrology Department, Biobizkaia Health Research Institute, Hospital Universitario Cruces, University of the Basque Country (UPV/EHU), CIBERDEM, CIBERER, EndoERN, Barakaldo, Bizkaia, Spain; ^3^Biobizkaia Health Research Institute, Hospital Universitario Cruces, University of the Basque Country (UPV/EHU), CIBERDEM, CIBERER, EndoERN, Barakaldo, Bizkaia, Spain; ^4^Endocrinology and Nutrition Department, Biobizkaia Health Research Institute, Hospital Universitario Cruces, EndoERN, Barakaldo, Bizkaia, Spain; ^5^Pediatric Endocrinology Department, Biobizkaia Health Research Institute, Hospital Universitario Cruces, EndoERN, Barakaldo, Bizkaia, Spain; ^6^Pediatric Endocrinology Department, Biobizkaia Health Research Institute, Hospital Universitario Cruces, CIBERDEM, CIBERER, EndoERN, Barakaldo, Bizkaia, Spain; ^7^Endocrinology and Nutrition Department, Biobizkaia Health Research Institute, Hospital Universitario Cruces, University of the Basque Country (UPV/EHU), CIBERDEM, CIBERER, EndoERN, Barakaldo, Bizkaia, Spain

**Keywords:** NGS, calcium, hypercalcemia, hypocalciuria, hyperparathyroidism

## Abstract

**Introduction:**

The disorders in the metabolism of calcium can present with manifestations that strongly suggest their diagnosis; however, most of the time, the symptoms with which they are expressed are nonspecific or present only as a laboratory finding, usually hypercalcemia. Because many of these disorders have a genetic etiology, in the present study, we sequenced a selection of 55 genes encoding the principal proteins involved in the regulation of calcium metabolism.

**Methods:**

A cohort of 79 patients with hypercalcemia were analyzed by next-generation sequencing.

**Results:**

The 30% of our cohort presented one pathogenic or likely pathogenic variant in genes associated with hypercalcemia. We confirmed the clinical diagnosis of 17 patients with hypocalciuric hypercalcemia (pathogenic or likely pathogenic variants in the *CASR* and *AP2S1* genes), one patient with neonatal hyperparathyroidism (homozygous pathogenic variant in the *CASR* gene), and another patient with infantile hypercalcemia (two pathogenic variants in compound heterozygous state in the *CYP24A1* gene). However, we also found variants in genes associated with primary hyperparathyroidism (*GCM2*), renal hypophosphatemia with or without rickets (*SLC34A1*, *SLC34A3*, *SLC9A3R1*, *VDR*, and *CYP27B1*), DiGeorge syndrome (*TBX1* and *NEBL*), and hypophosphatasia (*ALPL*). Our genetic study revealed 11 novel variants.

**Conclusions:**

Our study demonstrates the importance of genetic analysis through massive sequencing to obtain a clinical diagnosis of certainty. The identification of patients with a genetic cause is important for the appropriate treatment and identification of family members at risk of the disease.

## Introduction

The third cause of consultation with the endocrinologist, after diabetes and thyroid diseases, is the disorders in the metabolism of calcium. A dynamic balance between intestinal absorption, bone resorption, and renal excretion maintains the calcium metabolism. Calcium is required for many intracellular functions (signal transmission and many enzymatic reactions) and extracellular functions (blood coagulation, muscle contraction, nerve conduction, hormone release, and mineralization of bone) and is regulated primarily by the actions of vitamin D and parathyroid hormone (PTH). Furthermore, calcitonin, a hormone that is produced and released by the C cells of the thyroid gland, opposes the actions of the PTH by decreasing calcium levels in blood (1). The calcium-sensing receptor (CaSR) plays a central role in the regulation of extracellular calcium homeostasis. Thus, in the presence of a high extracellular calcium concentration [Ca^+2^], the activation of CaSR inhibits the secretion of PTH, whereas the effect is reversed under low [Ca^+2^] (2). Moreover, hyperphosphatemia, hypomagnesemia, and the adrenergic action also contribute to PTH secretion. On the other hand, the 1,25-dihydroxyvitamin D3 inhibits PTH secretion.

The hypercalcemia can be an incidental finding, but, sometimes, patients have symptoms as lethargy, hypotonia, anorexia, weight loss, polyuria, polydipsia, vomiting, abdominal pain, and constipation. In long-term or severe cases, kidney failure, pancreatitis, arrhythmias, seizures, and psychiatric condition could be present. Because many of these disorders have a genetic etiology, in the present study, we sequenced a selection, carried out in 2017, of 55 genes encoding the principal proteins involved in the regulation of calcium metabolism to perform a genetic characterization of a cohort of 79 patients diagnosed with hypercalcemia.

## Materials and methods

### Ethics statement

The study was approved by the Ethics Committee for Clinical Research of Euskadi (CEIC-E, code E20/31). A written informed consent was obtained from the patients, as well as their participating relatives and minors’ legal guardian for the publication of any potentially identifiable images or data included in this article. Patients and their participating relatives provided a written informed consent for the genetic study. The research was carried out in accordance with the Declaration of Helsinki on human experimentation of the World Medical Association.

### Patients

A cohort of 79 patients with hypercalcemia from 79 different unrelated families was included in this study. Patients were referred to our laboratory between 2003 and 2023. The main reason for this study was the genetic analysis of patients clinically diagnosed with familial hypocalciuric hypercalcemia (66 patients). Moreover, we included 13 patients who had neonatal or familial hypercalcemia. Patients with primary hyperparathyroidism were excluded. First-degree family members (mother and father) in 18 families with a genetic variant were analyzed. Clinical diagnoses were made by adult and pediatric endocrinologists or nephrologists working in 25 different hospitals. The molecular analysis was done in the Molecular Genetic Laboratory at Biobizkaia Health Research Institute, Barakaldo, Spain.

### Gene selection and DNA analysis

A selection of 55 genes identified as potentially associated with disorders of calcium metabolism was screened for pathogenic variants (**Table 1**). For next-generation sequencing (NGS), a targeted panel was designed using the computer tool Ion AmpliSeq Designer (Thermo Fisher Scientific, Waltham, Massachusetts, USA) with an expected coverage of 98%. This panel included the exon regions and flanking intronic (at least 40 bases) sequences of the 55 genes.

**Table 1:** Genes involved in the regulation of calcium metabolism included in the panel for analysis.

OMIM^®^	Gene	Phenotype
*139320	*GNAS*	Pseudohypoparathyroidism, type IA
*603666	*STX16*	Pseudohypoparathyroidism, type IB
*188830	*PRKAR1A*	Acrodysostosis 1, with or without hormone resistance
*600129	*PDE4D*	Acrodysostosis 2, with or without hormone resistance
*168468	***PTHR1***	Metaphyseal chondrodysplasia, Murk Jansen type
*123805	*PDE3A*	Hypertension and brachydactyly syndrome
*168470	*PTHLH*	Brachydactyly, type E2
*123810	*CREB1*	Histiocytoma, angiomatoid fibrous, somatic
*605314	*HDAC4*	Neurodevelopmental disorder with central hypotonia and dysmorphic facies. Primary hyperparathyroidism
*604386	*TRPS1*	Trichorhinophalangeal syndrome, type I, III
*608177	*EXT1*	Exostoses, multiple, type 1
*142989	*HOXD13*	Brachydactyly. Syndactyly. Synpolydactyly 1
*102576	*ACVR1*	Fibrodysplasia ossificans progressiva
*601756	*GALNT3*	Tumoral calcinosis, hyperphosphatemic, familial, 1
*601199	***CASR***	Hypocalciuric hypercalcemia, type I
*139313	***GNA11***	Hypocalciuric hypercalcemia, type II
*602242	***AP2S1***	Hypocalciuric hypercalcemia, type III
*613733	***MEN1***	Multiple endocrine neoplasia 1
*116899	*CDKN1A*	Multiple endocrine neoplasia 1
*600778	***CDKN1B***	Multiple endocrine neoplasia, type IV
*600431	*CDKN2B*	Multiple endocrine neoplasia 1
*603369	*CDKN2C*	Multiple endocrine neoplasia 1
*164761	***RET***	Multiple endocrine neoplasia IIA
*607393	***CDC73***	Hyperparathyroidism-jaw tumor syndrome
*603716	***GCM2***	Hyperparathyroidism 4
*168461	***CCND1***	Sporadic primary hyperparathyroidism
*605555	*AIP*	Pituitary adenoma 1, multiple types
*116806	***CTNNB1***	Sporadic primary hyperparathyroidism
*601573	***EZH2***	Weaver syndrome, sporadic primary hyperparathyroidism
*314980	***ZFX***	Sporadic primary hyperparathyroidism
*609506	*CYP27B1*	Vitamin D–dependent rickets, type I
*601769	*VDR*	Rickets, vitamin D–resistant, type IIA
*608713	*CYP2R1*	Rickets due to defect in vitamin D 25-hydroxylation deficiency
*126065	***CYP24A1***	Hypercalcemia, infantile, 1
*168450	*PTH*	Hypoparathyroidism, familial isolated 1
*602054	*TBX1*	DiGeorge syndrome
*605491	*NEBL*	DiGeorge síndrome, type 2
*607358	*AIRE*	Autoimmune polyendocrinopathy syndrome, type I, with or without reversible metaphyseal dysplasia
*131320	*GATA3*	Hypoparathyroidism, sensorineural deafness, and renal dysplasia
*604934	*TBCE*	Hypoparathyroidism-retardation-dysmorphism syndrome
*615292	*FAM111A*	Gracile bone dysplasia
*313430	*SOX3*	Panhypopituitarism, X-linked
*600980	*DMP1*	Hypophosphatemic rickets
*605380	*FGF23*	Hypophosphatemic rickets, autosomal dominant
*609826	***SLC34A3***	Hypophosphatemic rickets with hypercalciuria
*300550	*PHEX*	Hypophosphatemic rickets, X-linked dominant
*182309	***SLC34A1***	Nephrolithiasis/osteoporosis, hypophosphatemic, 1
*604990	*SLC9A3R1*	Nephrolithiasis/osteoporosis, hypophosphatemic, 2
*173335	*ENPP1*	Hypophosphatemic rickets, autosomal recessive, 2
*607009	*TRPM6*	Hypomagnesemia 1, intestinal
*603959	*CLDN16*	Hypomagnesemia 3, renal
*610036	*CLDN19*	Hypomagnesemia 5, renal, with ocular involvement
*171760	***ALPL***	Hypophosphatasia
*601814	*FXYD2*	Hypomagnesemia 2, renal
*607803	*CNNM2*	Hypomagnesemia 6, renal

Genes marked in bold are associated with hypercalcemia. *, indicates that the entry corresponds to a gene.

Genomic DNA was extracted from peripheral blood leukocytes using the MagPurix instrument (Zinexts Life Science Corp., New Taipei City, Taiwan, R.O.C.). DNA purity and concentration were then determined using Qubit 2.0 fluorometer (Thermo Fisher Scientific). Library preparation was done using the Ion AmpliSeq Library Kit v2.0 (Thermo Fisher Scientific) according to the manufacturer’s instructions. Samples were then sequenced using the Ion GeneStudio S5 System (Thermo Fisher Scientific). Base calling, read filtering, alignment to the reference human genome GRCh37/hg19, and variant calling were done using Ion Torrent Suite and Ion Reporter Software (Thermo Fisher Scientific). Not appropriately covered amplicons (<20×) and candidate variants were assessed by Sanger sequencing after polymerase chain reaction, sequenced with fluorescent dideoxynucleotides (BigDye Terminator v3.1 Cycle Sequencing Kit, Life Technologies, Grand Island, NY, USA), and loaded onto an ABI3130xl Genetic Analyzer (Thermo Fisher Scientific).

In order to confirm the deletion detected by NGS in the *TBX1* gene, a commercially available MLPA (Multiplex Ligation-dependent Probe Amplification) kit, SALSA MLPA Probemix P250 DiGeorge (MRC Holland, Amsterdam, The Netherlands), was used.

Novel DNA variants were named according to the Human Genome Variation Society guidelines (www.hgvs.org) and classified according to ACMG-AMP (American College of Medical Genetics and Genomics and the Association for Molecular Pathology) guidelines (3).

## Results

Thirty percent of our cohort (24 out of the 79 index cases) presented one pathogenic or likely pathogenic variant in genes associated with hypercalcemia. In total, we found 15 pathogenic variants, nine likely pathogenic variants, and 12 variants of uncertain significance (10 variants in patients with hypocalciuric hypercalcemia and two variants in patients with hypercalcemia). Importantly, our genetic study revealed 11 variants not described so far (**Table 2**).

**Table 2:** Molecular results in patients with a genetic variant.

Patient	Clinical Diagnosis	Genes affected	Variants	Form	Relatives*	Comments	Variant class+	Reference
CA0109	Hypercalcemia	*CASR*	NM_000388.4: c.1942C>T; p.Arg648*	Heterozygous	Father had the variant and hypercalcemia.	–	Pathogenic	(4)
CA0116	Hypocalciuric hypercalcemia	*CASR*	NM_000388.4: **c.2113_2115delGTG; p.(Val705del)**	Heterozygous	–	**ACMG criteria: PM1, moderate; PM2, moderate; PM4, moderate**	Likely pathogenic	**This study**
CA0115	Hypocalciuric hypercalcemia	*CASR*	NM_000388.4: c.659G>A; p.Arg220Gln	Heterozygous	Mother had the variant and hypercalcemia.	–	Pathogenic	(5)
CA0125	Hypocalciuric hypercalcemia	*CASR*	NM_000388.4: **c.2525T>C; p.(Leu842Pro)**	Heterozygous	Mother had the variant and hypercalcemia.	**ACMG criteria: PM1, moderate; PM2, moderate; PP2, supporting; PP3, moderate**	Likely pathogenic	**This study**
CA0022	Hypocalciuric hypercalcemia	*CASR*	NM_000388.4: c.164C>T; p.Pro55Leu	Mosaic	*de novo* variant	–	Pathogenic	(6)
CA0092	Hypocalciuric hypercalcemia	*CASR*	NM_000388.4: c.164C>T; p.Pro55Leu	Heterozygous	–	–	Pathogenic	(6)
CA0131	Hypocalciuric hypercalcemia	*CASR*	NM_000388.4: c.2738C>T; p.Ser913Phe	Heterozygous	–	–	Uncertain significance	rs751273631
CA0100	Hypocalciuric hypercalcemia	*CASR*	NM_000388.4: **c.254C>T; p.(Pro85Leu)**	Heterozygous	Mother had the variant and hypercalcemia.	**ACMG criteria: PM1, supporting; PM2, moderate; PP2, supporting; PP1, moderate**	Likely pathogenic	**This study**
CA0110	Neonatal hyperparathyroidism	*CASR*	NM_000388.4: **c.121C>A; p.(His41Asn)**	Homozygous	Both father and mother had the variant in heterozygous state and hypercalcemia.	**ACMG criteria: PM1, moderate; PM2, moderate; PP1, strong; PP2, supporting; PP3, supporting**	Pathogenic	**This study**
CA0093	Hypocalciuric hypercalcemia	*CASR*	NM_000388.4: c**.511A>G; p.(Ser171Gly)**	Heterozygous	Mother had the variant and hypercalcemia.	**ACMG criteria: PM1, moderate; PM2, moderate; PP1, moderate; PP2, supporting; PP3, supporting**	Likely pathogenic	**This study**
CA0147	Hypocalciuric hypercalcemia	*CASR*	NM_000388.4: **c.2912_2913delGCinsTT; p.(Gly971Val)**	Heterozygous	*de novo* variant.	**ACMG criteria: PS2, strong; PM2, moderate; PP2, supporting**	Likely pathogenic	**This study**
CA0133	Hypocalciuric hypercalcemia	*CASR*	NM_000388.4: c.1711G>T; p.Gly571Trp	Heterozygous	–	–	Pathogenic	(7)
CA0144	Hypocalciuric hypercalcemia	*CASR*	NM_000388.4: c.1906A>T; p.(Lys636*)	Heterozygous	–	**ACMG criteria: PVS1, strong; PM2, moderate**	Likely pathogenic	**This study**
								
CA0157	Hypocalciuric hypercalcemia	*CASR*	NM_000388.4: c.1394G>A; p.Arg465Gln	Heterozygous	Mother had the variant and hypercalcemia.	–	Pathogenic	(8)
CA0163	Hypocalciuric hypercalcemia	*CASR*	NM_000388.4: **c.1465T>C; p.(Tyr489His)**	Heterozygous	Mother had the variant and hypercalcemia.	**ACMG criteria: PM2, moderate; PP1, supporting; PP2, supporting; PP3, strong**	Likely pathogenic	**This study**
CA0096	Hypocalciuric hypercalcemia	*AP2S1*	NM_004069.3: c.43C>T; p.Arg15Cys	Heterozygous	Mother had the variant and hypercalcemia.	–	Pathogenic	(9)
CA0043	Hypocalciuric hypercalcemia	*AP2S1*	NM_004069.3: c.43C>T; p.Arg15Cys	Heterozygous	–	–	Pathogenic	(9)
CA0059	Hypocalciuric hypercalcemia	*AP2S1*	NM_004069.3: c.43C>T; p.Arg15Cys	Heterozygous	–	–	Pathogenic	(9)
CA0014	Hypercalcemia	*AP2S1*	NM_004069.3: c.44G>T; p.Arg15Leu	Heterozygous	*de novo* variant.	–	Pathogenic	(9)
CA0159	Hypocalciuric hypercalcemia	*VDR*	NM_001017536.2: c.1271C>G();306G>T; p.(Pro424Arg)();(Met102Ile)	Heterozygous/heterozygous	–	–	Uncertain significance/uncertain significance	rs200556498/rs200041268
CA0102	Hypercalcemia	*GCM2*	NM_004752.3: c.139C>T; p.Arg47Cys	Heterozygous	–	Associated with hyperparathyroidism	Uncertain significance	(10)
CA0143	Hypocalciuric hypercalcemia	*GCM2*	NM_004752.3: c.1003C>T; p.Pro335Ser	Heterozygous	–	–	Uncertain significance	rs1260165935
CA0048	Hypocalciuric hypercalcemia	*SLC9A3R1*	NM_004252.4: c.809G>A; p.Arg270His	Heterozygous	–	–	Uncertain significance	rs777978291
CA0104	Hypocalciuric hypercalcemia	*SLC9A3R1*	NM_004252.4:**c.107G>A; p.(Gly36Asp)**	Heterozygous	Mother had the variant but asymptomatic.	**ACMG criteria: PM2, moderate**	Uncertain significance	**This study**
CA0029	Hypocalciuric hypercalcemia	*CYP24A1*	NM_000782.5: c.469C>T; p.Arg157Trp	Heterozygous	–	Conflicting classifications of pathogenicity	Uncertain significance	(11)
CA0139	Infantile hypercalcemia	*CYP24A1*	NM_000782.5: c.[443T>C];[1186C>T]/p.[Leu148Pro];[Arg396Trp]	Compound heterozygous	Father had the c.443T>C variant and mother had the c.1186C>T variant, both asymptomatic.	–	Pathogenic/pathogenic	(12, 13)
CA0075	Hypocalciuric hypercalcemia	*CLDN19*/*CYP27B1*	NM_148960.2: c.59G>A; p.Gly20Asp/NM_000785.3: c.40C>T; p.Arg14Cys	Heterozygous/heterozygous	–	–	Pathogenic/uncertain significance	(14)/rs150648140
CA0112	Hypercalcemia	*SLC34A3*	NM_080877.2; c.232G>A; p.Gly78Arg	Heterozygous	Father had the variant but asymptomatic		Pathogenic	(15)
CA0065	Hypercalcemia	*SLC34A3*	NM_080877.2: c.586G>A; p.Gly196Arg	Heterozygous	Father had the variant but asymptomatic	–	Pathogenic	(16)
ME0136	Hypercalcemia	*SLC34A3*	NM_080877.2: c.1727G>T; p.Ser576Ile	Heterozygous	Father had the variant but asymptomatic	–	Uncertain significance	rs200090657
SOR0259	Hypercalciuric hypercalcemia	*SLC34A1*	NM_003052.4: c.272_292del21; p.Val91_Ala97del	Heterozygous	Mother with nephrolithiasis had this small deletion.	This variant alters the activity of the protein.	Likely pathogenic	(17)
CA0086	Hypocalciuric hypercalcemia	*SLC34A1*	NM_003052.4: c.1223T>A; p.Val408Glu	Heterozygous	–	This variant has been associated with idiopathic infantile hypercalcemia.	Pathogenic	(18)
CA0121	Hypocalciuric hypercalcemia	*ALPL*	NM_000478.5: **g.(?_21835231)_(21904741_)?dup**	Heterozygous	–	**Whole-gene duplication**	Uncertain significance	**This study**
CA0128	Hypocalciuric hypercalcemia	*ALPL*	NM_000478.5: **c.1097C>T; p.(Thr366Ile)**	Heterozygous	–	**ACMG criteria: PM1, moderate; PM2, moderate; PP2, supporting; PP3, supporting**	Likely pathogenic	**This study**
CA0080	Hypocalciuric hypercalcemia	*TBX1*	NM_080647.1: g.(?_19241636)_(21349222_)?del+	Heterozygous	*de novo* variant	Human 22q11.2 region: LCR22A-LCR22D. DiGeorge region (genes deleted: *CLTCL1*, *HIRA*, *CDC45*, *CLDN5*, *GP1BB*, *TBX1*, *TXNRD2*, *DGCR8*, *ZNF74*, *KLHL22*, *MED15*, *SNAP29*, and *LZTR1*).	Pathogenic	(19)
CA0068	Hypocalciuric hypercalcemia	*NEBL*	NM_006393.2: c.267C>G; p.Try89*	Heterozygous	–	–	Uncertain significance	rs147622517

Variants marked in bold have not been reported to date. *First-degree family members (mother and father) in 18 families were analyzed. +Classification according to ACMG-AMP guidelines (3). ACMG criteria: PVS, very strong evidence of pathogenicity; PS, strong evidence of pathogenicity; PM, moderate evidence of pathogenicity; PP, supporting evidence of pathogenicity.

We confirmed the initial clinical diagnosis (biochemical data are shown in **Table 3**) of familial hypocalciuric hypercalcemia in 25% of the patients (17 out of the 66 patients had pathogenic or likely pathogenic variants in the *CASR* and *AP2S1* genes). Moreover, we confirmed the initial clinical diagnosis of neonatal hyperparathyroidism in one patient (index case CA0110 had a homozygous pathogenic variant in the *CASR* gene) and infantile hypercalcemia in another patient (index case CA0139 had two pathogenic variants in compound heterozygous state in the *CYP24A1* gene).

**Table 3:** Laboratory findings of index cases with a genetic variant.

Patient	Gene affected	Gender (female/male)	Age at diagnosis (years)	Total serum calcium (mmol/L)		Serum phosphate (mmol/L)	25-OH vitamin D3 (ng/mL)	PTH (pg/mL)	UCa/Cr (mg/mg)	UCa (mg/24h)	Associated clinical data
CA0109	*CASR*	M	0.3	3.69		1.58	25.6	11.7	1.3	NA	Constipation
CA0116	*CASR*	M	16	2.87		1.32	26.9	63	0.08	225	Asthenia, hyporexia
CA0115	*CASR*	F	12.6	2.99		1.16	8.6	65	0.01	NA	Asymptomatic
CA0125	*CASR*	F	8	2.84		1.32	26	64	0.02	NA	Asymptomatic
CA0022*	*CASR*	F	1	NA		NA	NA	NA	NA	NA	NA
CA0092	*CASR*	M	58	2.94		0.65	NA	75	0.08	127	Asymptomatic
CA0131	*CASR*	F	56	2.67		1.26	34	96	NA	95	Asymptomatic
CA0100	*CASR*	F	4	2.92		NA	25.2	20.3	0.05	NA	Failure to thrive
CA0110	*CASR*	M	0.1	5.56		NA	15.1	NA	NA	NA	Atrioventricular block
CA0093	*CASR*	M	2	2.89		1.26	35.3	30.6	NA	24.6	Constipation
CA0147	*CASR*	F	13	2.92		1.23	35	44.3	0.05	NA	Asymptomatic
CA0133	*CASR*	M	49	2.92		1.07	32.3	71.8	NA	78	Nephrolithiasis
CA0144	*CASR*	F	27	2.74		NA	28.1	72.2	0.01	NA	Asymptomatic
CA0157	*CASR*	M	16	3.07		1.07	17.9	30.1	NA	137.6	Asymptomatic
CA0163	*CASR*	M	0.7	2.99		1.84	NA	NA	NA	111	Asymptomatic
CA0096	*AP2S1*	M	15	3.12		1.07	45	83	0.03	NA	Failure to thrive, attention deficit hyperactivity disorder
CA0043*	*AP2S1*	F	22	NA		NA	NA	NA	NA	NA	NA
CA0059	*AP2S1*	F	9	2.94		1.06	20	62	0.06	NA	Asymptomatic
CA0014	*AP2S1*	M	1.5	3.09		1.32	41	29	0.2	31	Asymptomatic
CA0159	*VDR*	M	50	2.69		0.87	13.2	62	NA	80.4	Asymptomatic
CA0102*	*GCM2*	F	85	NA		NA	NA	NA	NA	NA	Asymptomatic
CA0143*	*GCM2*	F	75	NA		NA	NA	NA	Calcium/creatinine clearance ratio: 0.004 mmol/l	NA	NA
CA0048	*SLC9A3R1*	F	64	2.64		0.94	30.8	90	NA	NA	Asymptomatic
CA0104	*SLC9A3R1*	F	58	2.59		NA	NA	84	0.005	NA	NA
CA0029	*CYP24A1*	F	68	2.84		0.9	18.2	103	0.007	80	Asymptomatic
CA0139	*CYP24A1*	M	0.6	3.29		1.13	166	2.1	0.3	NA	Failure to thrive
CA0075	*CLDN19*/*CYP27B1*	F	42	2.54		0.97	31.1	85.5	0.009	190	Nephrolithiasis
CA0112	*SLC34A3*	M	0.1	3.02		1.74	78	18.7	0.6	NA	Asymptomatic
CA0065	*SLC34A3*	F	10.9	2.94		1.26	NA	NA	0.5	307	Asymptomatic
ME0136	*SLC34A3*	F	10.6	2.74		1.16	19	134	0.3	221	Asymptomatic
SOR0259	*SLC34A1*	F	0.1	2.79		2.55	19	17	1.2	NA	Asymptomatic
CA0086	*SLC34A1*	F	36	2.64		NA	42.3	45.1	0.015	115	Asymptomatic
CA0121	*ALPL*	M	13.4	2.69		NA	22.2	42	0.01	NA	Asymptomatic
CA0128	*ALPL*	F	75	2.62		0.74	30	122	0.01	141	Asymptomatic
CA0080*	*TBX1*	F	16	NA		NA	NA	52	Calcium/creatinine clearance ratio: 0.004 mmol/l	NA	Asymptomatic
CA0068	*NEBL*	F	64	2.64		NA	NA	140	NA	100	Asymptomatic

PTH, parathyroid hormone; U, urinary. Reference ranges: total serum calcium (2.12–2.54 mmol/L); serum phosphate (children: 1.29–2.26 mmol/L; adults: 0.9–1.45 mmol/L); 25-OH vitamin D3 (25–80 ng/mL); intact PTH (8–51 pg/mL); UCa/Cr (< 0.20 mg/mg); U.Ca (100–300 mg/24 h); NA, not available. *The patient had hypercalcemia, but we do not have the biochemical data.

As expected, most patients had suspected variants in the *CASR* gene (15 patients) or *AP2S1* gene (four patients) that have been associated with hypocalciuric hypercalcemia type 1 (Online Mendelian Inheritance in Man (OMIM), #145980) and hypocalciuric hypercalcemia type 3 (OMIM, #600740), respectively (biochemical data are shown in **Table 3**).

Two index cases (CA0143 and CA0102) had variants of uncertain significance in the *GCM2* gene, and gain-of function mutations in the *GCM2* gene cause autosomal dominant hyperparathyroidism type 4 (OMIM, #617343), a disorder characterized by hypercalcemia and elevated PTH secretion by parathyroid glands. These patients had hypercalcemia but not hyperparathyroidism. One of the limitations of our study is the lack of functional studies. Therefore, we cannot assure that the hypercalcemia observed is due to these variants.

Furthermore, many patients had variants in genes usually associated with hypophosphatemia. Thus, five patients had suspected variants in sodium/phosphate cotransporters. Three families (CA0112, CA0065, and ME0136) had variants in the *SLC34A3* gene, two previously described as pathogenic and one of uncertain significance. This gene encodes the sodium/phosphate cotransporter 2C, and pathogenic variants in this gene have been associated with hypophosphatemic rickets with hypercalciuria (OMIM, #241530). Moreover, index cases CA0086 and SOR0257 had a pathogenic variant (p.Val408Glu) and a likely pathogenic variant (p.Val91_Ala97del), respectively, in the *SLC34A1* gene (sodium/phosphate cotransporter 2A). Pathogenic variants in the *SLC34A1* gene have been associated with infantile hypercalcemia type 2 (OMIM, #616963) and nephrolithiasis/osteoporosis hypophosphatemic type 1 (OMIM, #612286). Another two patients (CA0048 and CA0104) had variants of uncertain significance in the *SLC9A3R1* gene. This gene encoded a sodium/hydrogen exchanger regulatory cofactor (NHERF1). Pathogenic variants in the *SLC9A3R1* gene have been associated with autosomal dominant nephrolithiasis/osteoporosis hypophosphatemic type 2 (OMIM, #612287).

On the other hand, we found variants in genes associated with vitamin D metabolism; one index case (CA0139) had two pathogenic variants in compound heterozygous (p.[Leu148Pro];[Arg396Trp]) in the *CYP24A1* gene that encodes the vitamin D 24-hydroxylase; in the same gene, index case CA0029 had a variant of uncertain significance (p.Arg157Trp) in heterozygous state. Pathogenic variants in the *CYP24A1* gene have been associated with infantile hypercalcemia type 1 (OMIM, #143880). Finally, in one patient (CA0159), we found two variants of uncertain significance in the *VDR* gene (vitamin D receptor). Unfortunately, we have not been able to check if the variants are on different alleles. Pathogenic variants in the *VDR* gene have been associated with autosomal recessive vitamin D dependent rickets type 2A (OMIM, #277440).

In addition, we found suspected variants in other five genes not associated with the phenotype (*TBX1*, *ALPL*, *NEBL*, *CLDN19*, and *CYP27B1*) (**Table 2**). Some of these variants were found in heterozygous state (*CLDN19* and *CYP27B1*), in diseases described previously as recessively inherited.

Regarding patients without a genetic diagnosis, more studies should be carried out, for example, exome or genome sequencing, because they presented phenotypic characteristics similar to patients with a genetic variant.

## Discussion

In this study, we examined the presence of genetic alterations in genes related to calcium metabolism in a cohort of 79 patients with hypercalcemia. We found pathogenic or likely pathogenic variants in 30% of patients in our cohort (24 out of 79 patients) that could explain the phenotype observed in those patients. Moreover, we have genetically confirmed the clinical diagnosis given by the clinician in the 24% of our cohort (17 patients with familial hypocalciuric hypercalcemia, one patient with neonatal hyperparathyroidism, and another patient with infantile hypercalcemia). Our complete genetic study revealed 15 pathogenic variants, nine likely pathogenic variants, and 12 of uncertain significance variants. Importantly, our genetic study revealed 11 novel variants.

We confirmed the clinical diagnosis of hypocalciuric hypercalcemia in 17 patients. Our high diagnostic yield respect others cohorts analyzed (20) may be due to a better clinical and biochemical characterization of the patients, to be restrict in the selection of the patients and the division in two groups (in one group, all the patients have hypercalcemia and hypocalciuria). Importantly, one of these patients, index case CA0022, had a *de novo* pathogenic variant in the *CASR* gene in mosaic (variant found in a 20% of reads), assuming that, it is possible that a minimal amount of defective protein is enough to develop the disease. To our knowledge, familial hypocalciuric hypercalcemia due to mosaicism in *CASR* has not been described before. Moreover, we found a pathogenic variant (p.Arg648*) in the *CASR* gene in another patient (index case CA0109) who had hypercalcemia but with hypercalciuria. It has been previously described that, in some cases, urinary calcium levels are normal or even high. Furthermore, the biochemical profile varies considerably and this variability is thought to be mutation dependent (21).

On the other hand, in four patients initially diagnosed with familial hypocalciuric hypercalcemia, we found suspected variants in other genes (*TBX1*, *NEBL*, and *ALPL*) not associated with the phenotype studied, and the definitive diagnosis could be changed. Thus, index case CA0080 had hypercalcemia, parathyroid hyperplasia, and hypocalciuria with a family history of hypocalciuria (mother and brother). Moreover, she presented with autoimmune hypothyroidism. In this patient, we found a pathogenic deletion in the DiGeorge syndrome chromosome region 22q11.2 (*TBX1* gene included). It is difficult to assess how this deletion is influencing the patient’s phenotype because DiGeorge syndrome comprises hypocalcemia and hypoparathyroidism with parathyroid hypoplasia (OMIM, #188400). Furthermore, her mother and brother with hypocalciuria do not have the deletion. It is important to note that some patients with the deletion have minimal clinical expression. Thus, it has been described that 10% to 25% of parents of patients with DiGeorge exhibit the deletion and have no symptoms (22), and hypocalcemia has been described in up to 80% of adults with a 22q11.2 deletion sometime during their lifetime (23). The patient could have another alteration because the hypocalciuria detected is inherited. Moreover, environmental factors such as hydration and sodium intake may explain why a patient with DiGeorge syndrome might have hypercalcemia. On the other hand, patient CA0080 presented an autoimmune disease, and this disease is observed in most age groups with this large deletion in the chromosome region 22q11.2 (24). In another study, the authors described the phenotypic features of 78 adults with this deletion, and 20.5% presented with hypothyroidism (25). Importantly, another index case with hypocalciuric hypercalcemia (CA0068) of our cohort had a nonsense variant of uncertain significance (p.Try89*) in the *NEBL* gene. The *NEBL* gene has been found deleted in two patients with DiGeorge syndrome type 2, who showed cardiac defects, but not in two patients with the more distal deletion, which is associated with hypoparathyroism, deafness, and renal dysplasia (26).

Among patients with hypocalciuric hypercalcemia, two index cases (CA0121 and CA0128) had novel variants in the *ALPL* gene (one large duplication and one missense, respectively). The *ALPL* gene is known to cause autosomal recessive infantile hypophosphatasia (OMIM, #241500) and dominant or recessive adult odontohypophosphatasia (OMIM, #146300). However, index case CA0121 had high levels of alkaline phosphatase (655 IU/L; normal range, 44 IU/L to 147 IU/L). Therefore, as far as we know, this duplication could be the first variant reported in the *ALPL* gene with a gain of function effect and associated with high levels of alkaline phosphatase. He had the whole-gene *ALPL* duplicated, and this is the first whole-gene duplication detected in literature. On the other hand, patient CA0128 had osteopenia and weakness of the femoral head, clinical characteristic of hypophosphatasia. Unfortunately, we do not have the alkaline phosphatase value of patient CA0128. Administration of drugs that inhibit bone resorption is contraindicated in patients with hypophosphatasia. She was treated with vitamin D, which could have caused hypercalcemia in this patient. Further studies aimed at the functional characterization of these variants will be of help in defining the hypothesized pathogenic roles of these two variants in the *ALPL* gene.

Seven patients diagnosed with hypercalcemia had a variant in heterozygous state in genes associated with hypophosphatemia (*SLC34A3*, *SLC34A1*, and *SLC9A3R1* genes). Minor symptoms such as mild hypophosphatemia and bone demineralization over time or kidney stones have been described in patients carrying heterozygous variants in these genes (27). Probably, in these patients, hypercalcemia may be justified because the renal phosphate wasting activates the secretion of calcitriol, which, in turn, increases calcium intestinal absorption (28). Moreover, we identified a heterozygous variant in the *CYP24A1* gene in one patient (CA0029) with hypocalciuric hypercalcemia. Figueres et al. found the p.(Arg157Trp) variant in compound heterozygous state with a second potentially causative variant in two patients with hypercalcemia (11). On the other hand, allele frequency is greater than expected for disorder (0.33% of alleles in individuals of European descent in Genome Aggregation Database). Due to the absence of functional analysis the clinical significance of this variant is uncertain. Although an autosomal dominant disease inheritance has been proposed (29), we cannot exclude a second undetected variant in this patient. We recommended studying the genes associated with genetic rickets (*SLC34A1*, *SLC34A3*, and *SLC9A3R1*) in those patients with hypercalcemia of suspected genetic cause but without a confirmatory genetic diagnosis, because they could have a heterozygous pathogenic variant.

Finally, one patient (CA0159) had two variants of uncertain significance in the *VDR* gene. Although, patient CA0159 had hypophosphatemia and high PTH levels, it does not present the typical characteristics of the disease such as rickets, alopecia, cutaneous cysts, or hypocalcemia. Therefore, these two variants are probably located on the same allele or have no clinical relevance.

In conclusion, our study shows the utility of NGS in unraveling the genetic origin of disorders in the calcium and phosphorus metabolism and reveled interesting findings that demonstrate the importance of genetic analysis through massive sequencing (panels, exome, and genome) to obtain a clinical diagnosis of certainty. The identification of patients with a genetic cause is important for the appropriate treatment and identification of family members at risk of the disease.

## Calcium and phosphorus metabolism molecular biology group

Idoia Martínez de LaPiscina, Laura Saso, Mirian Sánchez, Blanca González, Amaia Rodríguez, Olaia Velasco, Josu Aurrekoetxea, Begoña Calvo, Leire Gondra, June Corcuera, Ainhoa Camille Aranaga, Candela Baquero (Biobizkaia Health Research Institute, Hospital Universitario Cruces, Barakaldo, Bizkaia, Spain), María López-Iglesias (Endocrinology Department, Hospital General La Mancha Centro, Ciudad Real, Spain), Julia Sastre, María Ángeles Fernández (Hospital Virgen de la Salud, Toledo, Spain), Marta Murillo (UAB, Servei de Pediatria, Unitat d’Endocrinologia Pediàtrica, Hospital Universitari Germans Trias i Pujol, Badalona, Spain), Laura Martínez, Héctor Galbis, Luz Martínez, María Soledad Marín (Hospital Rafael Méndez, Lorca, Spain), Carla Criado, María Isidoro (Unidad de Referencia Regional de Enfermedades Raras DiERCyL, Centro de Referencia Nacional de Cardiopatías Familiares CSUR, Complejo Asistencial Universitario de Salamanca, Salamanca, Spain), Tommaso Matteucci, Ismene Bilbao (Hospital Universitario Donostia, Donostia, Spain), Javier Lumbreras, Diego de Sotto, Jordi Rosell (Hospital Universitario Son Espases, Palma de Mallorca, Spain), Rosa María Sánchez-Dehesa (Hospital Universitario Príncipe de Asturias, Madrid, Spain), María Pilar Bahillo (Hospital Clínico Universitario de Valladolid, Valladolid, Spain), Juan Cruz Len (Hospital de Txagorritxu, Vitoria, Spain), Raquel Barrio (Hospital Universitario Ramón y Cajal, Madrid, Spain), Sara Marsal, Olga Simó Servat, Gema Ariceta (Hospital Universitario Vall d’Hebron, Barcelona, Spain), Raúl Calzada (Instituto Nacional de Pediatría, Ciudad de México, México), Carmen Lourdes Rey Cordo (Pediatric Endocrinology Department, Hospital Álvaro Cunqueiro, EOXI, Vigo, Spain), Juan Marín (Hospital Clinic Universitari, Valencia, Spain), Jesús Barreiro (Hospital de Santiago de Compostela, Santiago de Compostela, Spain), Joaquín Ramírez (Hospital Príncipe de Asturias, Madrid, Spain), Jessica Ares, Cecilia Sánchez (Hospital Universitario Central de Asturias, Oviedo, Spain), Dulce Calderón (Hospital Virgen de la luz, Cuenca, Spain), Esteban Jodar (Hospital Quirón, Madrid, Spain), Jesús Manuel Morán (Hospital Virgen del Puerto, Plasencia, Spain), Cristina Castro (Hospital Universitari Doctor Peset, Valencia, Spain), José Manuel Rial (Hospital Universitario Hospiten Rambla, Santa Cruz de Tenerife, Spain) and Teresa Molins (Hospital Universitario de Navarra, Navarra, Spain).

## Data availability statement

The data presented in the study are deposited in the ClinVar repository, accession numbers SCV004708174 - SCV004708203 and SCV004708204 -- SCV004708209.

## Ethics statement

The studies involving humans were approved by Ethics Committee for Clinical Research of Euskadi (CEIC-E, code E20/31). The studies were conducted in accordance with the local legislation and institutional requirements. Written informed consent for participation in this study was provided by the participants’ legal guardians/next of kin.

## Author contributions

AG: Conceptualization, Formal analysis, Funding acquisition, Investigation, Methodology, Validation, Writing – original draft, Writing – review & editing. LM: Conceptualization, Funding acquisition, Investigation, Visualization, Writing – original draft, Writing – review & editing. SG: Formal analysis, Investigation, Methodology, Validation, Writing – original draft, Writing – review & editing. PG: Investigation, Resources, Validation, Visualization, Writing – review & editing. GG: Investigation, Resources, Validation, Visualization, Writing – review & editing. IR: Investigation, Resources, Validation, Visualization, Writing – review & editing. Gd: Data curation, Formal analysis, Investigation, Methodology, Writing – review & editing. AD: Methodology, Writing – review & editing. AA: Investigation, Resources, Validation, Writing – review & editing. RM: Investigation, Validation, Visualization, Writing – review & editing. IU: Investigation, Validation, Visualization, Writing – review & editing. SG: Conceptualization, Data curation, Validation, Visualization, Writing – original draft, Writing – review & editing. LC: Conceptualization, Data curation, Funding acquisition, Investigation, Resources, Validation, Writing – original draft, Writing – review & editing.
