# Correction: Genetic profile of a large Spanish cohort with hypercalcemia

**DOI:** 10.3389/fendo.2025.1730898

**Published:** 2025-11-14

**Authors:** Alejandro García-Castaño, Leire Madariaga, Sara Gómez-Conde, Pedro González, Gema Grau, Itxaso Rica, Gustavo Pérez de Nanclares, Ana Belén De la Hoz, Aníbal Aguayo, Rosa Martínez, Inés Urrutia, Sonia Gaztambide, Calcium Phosphorus Metabolism Molecular Biology Group, Luis Castaño

**Affiliations:** 1 Biobizkaia Health Research Institute, Hospital Universitario Cruces, CIBERDEM, CIBERER, EndoERN, Barakaldo, Bizkaia, Spain; 2 Pediatric Nephrology Department, Biobizkaia Health Research Institute, Hospital Universitario Cruces, University of the Basque Country (UPV/EHU), CIBERDEM, CIBERER, EndoERN, Barakaldo, Bizkaia, Spain; 3 Biobizkaia Health Research Institute, Hospital Universitario Cruces, University of the Basque Country (UPV/EHU), CIBERDEM, CIBERER, EndoERN, Barakaldo, Bizkaia, Spain; 4 Endocrinology and Nutrition Department, Biobizkaia Health Research Institute, Hospital Universitario Cruces, EndoERN, Barakaldo, Bizkaia, Spain; 5 Pediatric Endocrinology Department, Biobizkaia Health Research Institute, Hospital Universitario Cruces, EndoERN, Barakaldo, Bizkaia, Spain; 6 Pediatric Endocrinology Department, Biobizkaia Health Research Institute, Hospital Universitario Cruces, CIBERDEM, CIBERER, EndoERN, Barakaldo, Bizkaia, Spain; 7 Endocrinology and Nutrition Department, Biobizkaia Health Research Institute, Hospital Universitario Cruces, University of the Basque Country (UPV/EHU), CIBERDEM, CIBERER, EndoERN, Barakaldo, Bizkaia, Spain

**Keywords:** NGS, calcium, hypercalcemia, hypocalciuria, hyperparathyroidism

An incorrect **Funding** statement was provided. The **Funding** statement for the Institute of Health Carlos III was displayed as “Institute of Health Carlos III (PI21/01419)” The correct **Funding** statement reads: “The author(s) declare financial support was received for the research, authorship, and/or publication of this article. This work was supported by Project PI21/01419, funded by the Instituto de Salud Carlos III and co-funded by European Union (ERDF “A way to make Europe”); the Department of Health (2019111052); and the Department of Education (IT-1281-19) of the Basque Government. This work is generated within the Endocrine European Reference Network (Project ID number of Endo-ERN: 739527). The funders had no role in study design, data collection and analysis, decision to publish, or preparation of the manuscript.”

The original version of this article has been updated.

